# Community perception of causes of death using verbal autopsy for diabetes mellitus in Saudi Arabia

**DOI:** 10.1371/journal.pgph.0001690

**Published:** 2023-12-05

**Authors:** Faleh Alyazidi, Deler Shakely, Max Petzold, Fawaz Alyazidi, Laith Hussain-Alkhateeb

**Affiliations:** 1 School of Public Health and Community Medicine, Institute of Medicine, Sahlgrenska Academy, University of Gothenburg, Gothenburg, Sweden; 2 Department of Public Health, College of Health Sciences at Al-Leith, Umm Al-Qura University, Al-Leith, Kingdom of Saudi Arabia; 3 Infectious Diseases Control Department, Executive Directorate of Preventive Medicine, Makkah Healthcare Cluster, Makkah, Kingdom of Saudi Arabia; 4 Population Health Research Section, King Abdullah International Medical Research Centre, Riyadh, Saudi Arabia; Johns Hopkins University Bloomberg School of Public Health, COLOMBIA

## Abstract

Diabetes mellitus is a serious global health issue which significantly impacts public health and socioeconomic development. Exploring how the community perceives the causes of death and their associated risk factors is crucial for public health. This study combines verbal autopsy (VA) with the Type 2 Diabetes Mellitus (T2DM) register to explore community perceptions of causes of death and associated influential factors in Makkah province, Saudi Arabia. 302 VA interviews were conducted with relatives or caregivers of deceased who died between 2018 and 2021 based on T2DM medical register from Alnoor Specialist Hospital in Makkah City, Saudi Arabia. Cause-specific mortality fractions (CSMFs) obtained from the VA using the InterVA-5 model were utilized to assess community perception. We used a multivariable logistic regression model to determine factors influencing community perceptions of causes of death. Lin’s CCC with 95% CI was used to analyze the concordance for the CSMFs from verbal autopsy causes of death (VACoD) as a presumed reference standard and family-reported causes of death (FRCoD). The outcomes of this study demonstrate a generally broad spectrum of community perceived mortalities, with some critical misconceptions based on the type of death and other vital events like marital status, with an overall CCC of 0.60 (95% CI: 0.20–1.00; p = 003). The study findings demonstrate that community perception is weak if the deceased was male compared to female (aOR: 0.52; 95% CI: 0.26–1.03) and if the deceased was > = 80 years compared to 34–59 years (aOR: 0.48; 95% CI: 0.16–1.38), but it significantly improves among married compared to single (aOR: 2.13; 95% CI: 1.02–4.42). Exploring community perception of causes of death is crucial as it provides valuable insights into the community’s understanding, beliefs, and concerns regarding mortality. Higher or lower community perception is attributed to how people may perceive risk factors associated with the causes of death, which can guide public health planning and interventional programs. The study findings further emphasize the need to employ robust and standardized VA methods within the routine medical services for a systemized assessment of families’ reported causes of death.

## Introduction

Diabetes mellitus (DM) remains a major global public health issue with a significant impact on public health and socio-economic development. Diabetes is one of the top ten causes of death worldwide. Together with cardiovascular diseases, respiratory diseases, and cancer, these conditions account for over 80% of all premature non-communicable diseases (NCDs) deaths [1]. The International Diabetes Federation estimates that 536.6 million people were living with diabetes in 2021, and this number is projected to increase by 46%, reaching 783.2 million in 2045, with approximately 50% of all diabetic individuals are unaware of their conditions [2]. Several reports have attributed the prevalence of DM in the Middle East and North Africa regions to the increased per capita income, urbanization, and pronounced lifestyle changes that enabled physical inactivity and increased the obesity rate [3,4]. In the context of chronic illnesses and treatment management, community perception–which is defined as “a combination of experiences, expectations, and perceived needs” [5]–is critical for public health administrative practices. A comprehensive and integrated approach to understanding how communities perceive health and disease is crucial for developing relevant and effective health intervention strategies and can empower individuals to seek and handle health knowledge properly [6–8]. Individuals are more likely to take action to prevent illnesses if they acknowledge their susceptibility to a particular form of illness or if they are likely to experience serious consequences [9]. Lay concepts of health and illness provide essential information to biomedical models [10,11] and can significantly impact communities’ health and illness behaviors, which requires systematic means of assessment at the population level [7,11].

Saudi Arabia is associated with this global epidemic of NCDs [12,13]. According to the World Health Organization (WHO) [14], Saudi Arabia has the second highest rate of diabetes in the Middle East (7^th^ highest in the world), with an estimated population of 7 million diabetics and more than 3 million pre-diabetics [15], which is a serious public health issue. Furthermore, in 2021, the International Diabetes Federation reported that the prevalence of diabetes in adults in Saudi Arabia stood at approximately 17.7% [16]. According to the Global Health Estimates provided by the WHO, diabetes emerged as a prominent cause of mortality in Saudi Arabia, accounting for 12% among males and 10.1% among females [14].

Previous Saudi reports have shown that the success of national response strategies is heavily dependent on the public’s perceptions and attitudes toward the risk of an epidemic and the importance of preventive measures [17]. Addressing the health literacy problems among a culturally diverged Saudi population will be crucial by virtue of the undergoing health system re-engineering towards the Saudi 2030 Vision. The Vision 2030 Health Sector Transformation Program was initiated in 2021 with a five-year goal of reforming the health sector to become a comprehensive, effective, and integrated health system that prioritizes the health of individual and society, including citizens, residents, and visitors [18]. A growing body of evidence has additionally demonstrated that inadequate health literacy is significant among the Saudi population [19–21], whereby negative perceptions of diabetes risk factors can impede disease management and prevention. Exploring how individuals make appropriate health decisions in various contexts, including their homes, workplaces, communities, healthcare systems, and political arenas, is a fundamental aspect of understanding the concept of “health literacy”[7,22]. To comprehensively assess health literacy, it is imperative to develop a deep understanding of how lay people perceive health, illness, and the causes of death [7]. Patients with poor health literacy skills are prevalent among the Saudi population. A published cross-sectional survey conducted in Saudi Arabia to describe the distribution of low health literacy in the Saudi population found that 46% of respondents were classified as having low health literacy; this was associated with older age groups [20]. This group demands actively engaged healthcare professionals to effectively simplify care management, which will only add more burden to the already overstretched health system caused by COVID-19 and other emerging diseases.

Global statistics show that about half of annual deaths pass without formal medical certification leaving crucial causes of death information outside any form of useful application for policy making and public health program monitoring [23–27]. While this issue is broadly associated with low- and middle-income countries (LMICs) due to insufficient investment in civil registration and vital statistics (CRVS) systems [27], higher income countries are also prone to misclassification of crucial international classification of disease (ICD) information [28]. Saudi Arabia is among countries facing demographic transition, which can influence doctor-patient communications and hence the ultimate uptake of health information and their classifications.

In the local context, Prior studies in Saudi Arabia have identified gaps in mortality data [29–32], particularly in causes of death recording and reporting. Saudi Arabia encounters significant challenges in the realm of death reporting, marked by divergent death certification practices across health service sectors in the absence of a standardized electronic reporting system [29]. Due to emergencies, administrative or cultural factors, it is often that ICDs are inconsistently assigned to medical certifications, which poses civil registration and vital statistics quality and completeness implications, according to more recent reports [29–31]. While the literature on Saudi civil registration and vital statistics and the estimated gaps of causes of death information is scarce, a recent study conducted during the first half of 2020 to examine demographic characteristics, patterns, determinants, and primary causes of death revealed a gap of around 20% of causes of deaths data [31].

Verbal autopsy (VA)–standardized interviews conducted by trained fieldworkers or healthcare staff with final caregivers or close relatives of the deceased on the medical signs, symptoms, and circumstances surrounding deaths–has emerged in more recent time as a widely used method for determining causes of death in settings where adequate reporting and reliable classification of causes of death are distracted [33,34]. While this set of VA information is classically processed by physicians to determine a likely medical cause of death, computerized tools such as *InterVA-5* are being used and can offer a consistent, faster, and cost-effective approach to assigning causes of death [7,35,36]. VA is commonly conducted within registered or unregistered population and can consistently elicit information through structured and narrative sections using the standardized WHO VA questionnaires. At the end of the VA interview, the interviewer would typically finish off with asking respondents about their own interpretation of the causes of death of their deceased, such information that can form the basis of understanding community perception of causes of death. VA is a standardized tool, which has been thoroughly validated for its validity and reliability across different settings and causes of death categories as a reliable source to fill in causes of death gaps [35,37]. Rural Saudi Arabia presumably faces greater challenges of inadequate causes of death register, coupled with poorer accessibility to healthcare facilities due to different circumstantial factors [31], essentially how people perceive ill-health and act accordingly. This VA method will likely facilitate a large-scale assessment and classification of causes of death information at the population level [23,26]. The year 2030 is expected to be an exceptional milestone for Saudi Arabia, with a comprehensive agenda involving the assessment of essential national and global goals, including the 17 Sustainable Development Goals (SDGs). However, gaps in registering vital data persist (a main task attributed to the CRVS), and despite increasing calls for employing standardized VA concepts to strengthen public health applications, including the CRVS, the VA approach is entirely novel in Saudi Arabia and many other neighboring countries where gaps in causes of death information exist. Exploring the perception of public health aspects, essentially how causes of death are perceived within communities in Saudi Arabia, is crucial while the country’s health system is now undergoing a significant re-engineering process towards national and global agenda. Within the Saudi research and health policy interests, no studies have sought standardized tools such as the VA to explore the community perception of causes of death, which can have significant implications in guiding public health planning and novel interventional programs. This study will employ VA to explore the community perceptions of causes of death based on T2DM register in Makkah province, Saudi Arabia. Physician-certified causes of death records will be re-assessed using VA and the derived VACoD will be presumed reference standard against the family-reported causes of death (FRCoD) for determining the community perception across different causes of death groups and population backgrounds and characteristics. Higher correlation between VACoD and FRCoD refers to an adequate perception of the community of corresponding causes of death based on the pre-validated standardized VA tool.

## Methodology

### Study population and context

A total of 1,609 deaths occurred between 2018 and 2021 were retrieved from the T2DM medical register at Alnoor Specialist Hospital. Of which, 302 verbal autopsy interviews were conducted with relatives or caregivers of the deceased. Alnoor Specialist Hospital–a public hospital in Makkah city, which provides a broad range of specialized services, including emergency, surgery, and other referral services to a population size of 2.5 million. All patients included in this study fulfilled the study criteria; death occurred between 2018 and 2021 (small history interval to reduce recall bias during the VA process), at least one clinical diagnosis of T2DM, and informed consent to perform the VA interview given by the respondents. Furthermore, in instances where respondents/relatives did not provide consent or refused to participate, or if the contact information of relatives/next of kin was incorrect or unreachable, they were excluded from the study.

Saudi Arabia, the largest nation in the Arabian Peninsula, encompasses approximately 2 million square kilometers and is administratively divided into 13 provinces. According to the data from the General Authority for Statistics, the overall population in Saudi Arabia reached 32,175,224 in 2022, with a gender distribution of 61.2% male and 38.8% female [38] Makkah is a large province and of a relatively dynamic population compared to other Saudi provinces, mainly due to their close and constant interaction with people who come from all over the world to perform religion pilgrimage (Hajj and Umrah). This frequent (short and long-term) stay of a large population-scale creates a special demographic mix where migration of culture, economy, diseases, and demography is highly prevalent in this region. This diverged community has the potential to influence the community perceptions and beliefs about a certain phenomenon, such as how they perceive illness or causes of death.

### Verbal autopsy interpretation

For VA interpretation, several software programs are used for automated interpretation such as *’Smart VA’*, *’InSilicoVA’*, and *’InterVA’* [39–41], with the *InterVA* being used more broadly [23,41]. The 2016 WHO VA instrument has been updated to contain all the input variables needed by all three software, making their use more consistent and standardized [36,42,43]. In recent years, a series of InterVA models have been created to interpret VA data using Bayesian probabilistic modeling [44]. In this study, VA data has been processed using InterVA-5 software to obtain the likelihood causes of death.

VA uses a Bayesian probability model to determine the most probable cause of death and their corresponding likelihoods for each VA case [6,34,37]. The Bayesian concept operation permits the combination of medical experts’ views as prior and relevant available data, which can collectively augment the model outputs [7,45]. This methodological process used in the InterVA model is explained in full elsewhere [44]. Using Bayes theorem, up to three probable causes of death (likelihood) are typically assigned for each case by the automated InterVA tool, given the specific signs and symptoms reported [34,41]. Each assigned cause has corresponding likelihoods, and the sum likelihoods of all assigned causes has a maximum value of 1.00 (or 100%). In cases where the likelihood proportions did not add up to 1.0 for a particular case, any remaining margin of likelihood not accounted for by the likelihood of the first, second, and third causes can therefore be included as a partial indeterminate component in analyzing overall cause of death and CSMFs patterns. It has been suggested that this is a more practical approach than aggregating the sum of small residual probabilities of unlikely causes, which might lead to misleading conclusion in a large number of cases [44]. The Cause-Specific Mortality Fraction (CSMF) is an output from the algorithm. CSMF corresponds to the proportion that each cause of death category contributed to the total number of deaths [6,7,45].

### Data collection

Information on the deceased’s background and characteristics were taken directly from the medical register at Alnoor Specialist Hospital. Death records contained data for capturing vital events [deaths in and out-hospital, date of death, sex, age, ICD-10 code of causes of death, and contact details of the next of kin]. Considering the heterogeneous and dynamic population in this Province, other characteristics of the deceased such as nationality, marital status, and education, were also considered. We selected participants from the Alnoor Specialist Hospital in Makkah City using a simple random sampling method. The sample size of (311) was calculated using the following formula [46].

$$\text{n}=\frac{{\text{NZ}}_{\left(1-\alpha /2\right)}^{2}*\text{P}(1-\text{P})}{{\text{d}}^{2}*(\text{N}-1)+{\text{z}}_{\left(1-\alpha /2\right)}^{2}*\text{P}(1-\text{P})}$$

Where N: Represents the total population for infinite correction; N = 1609.

Z (1-α/2): Denotes the standard value related to alpha error at 5% that equals to 1.96.

P: Represents the proportion of overall agreement or assumed CCC for community perception of causes of death (50%).

d: Signifies the confidence limit equals 5% (at a confidence interval of 95%).

A total sample of 302 relatives or close caregivers of the deceased were eventually consented to conduct the VA interview. In addition, interviews were administered by the first author together with a trained research assistant. The standard face-to-face VA interview process has been challenged by the COVID-19 pandemic, which coincided with the strict physical distancing preventive measures implemented in Saudi Arabia. Therefore, telephone interviews were sought as a substitute for the standard face-to-face method. The interviews were conducted in Arabic without audio-recording, but data were reviewed and safely stored at the university electronic databases after removing all personal identifiers, and all participants were anonymized. Closed-ended questions were collected using the Open Data Kit (ODK) on Android smartphones. VA data were reported into a Microsoft Access database, and the participants’ study IDs were used to link the VA data with the background and characteristic information.

### Data management

The deceased’s age was categorized into four age groups (34–59, 60–69, 70–79, and > = 80 years old), and the levels of education were grouped as *illiterate*, *basic education including (primary*, *secondary school*, *and high school)*, *and advanced education including* university level. The relationship between the VA informants and the deceased was classified into two groups (first-degree relative and second-degree relative), whereby the deceased marital status was dichotomized as single and married. The place of death was recorded and processed as binary information (home, hospital/health facility). The causes of death were further categorized into broader categories, described in S1 Table.

### Statistical analysis

Numerical and graphical descriptive statistics were generally sought for causes of death data in overall and per groups. Each individual VA was processed using InterVA-5 model, which assigns up to three likely causes of death per WHO VA cause categories [47], while FRCoDs were given as a single or two causes of death. Crude and adjusted logistic regression for the binomial agreement (agreement or not) between VACoD and FRCoD was analyzed against the informant’s background, characteristics, and socioeconomic factors for each cause of death category. All potential confounders from the background and characteristic factors were assessed in a forward-wise likelihood ratio test. Statistical analyses were performed using STATA release 17.0 software using a significant level of 5% as a cut-off for the hypothesis test.

Generated causes of death likelihoods were utilized to calculate population-level CSMFs for each cause of death group between VACoD and FRCoD, and their proportional absolute differences and 95% confidence intervals were calculated. A concordance correlation coefficient (CCC) for the CSMFs from VACoD and FRCoD was also calculated using Lin’s CCC with 95% CI [48]. Based on Pearson’s correlation, the CCC compares the viewpoints between the two measures–just like other correlation tests, CCC has a range of -1 to 1, with 1 representing perfect agreement [49]. The CCC combines precision and accuracy measures to determine how far the observed data vary from the perfect concordance line (that is, the line at 45 degrees on a square scatter plot). Lin’s coefficient increases in value as a function of the data’s reduced major axis’s closeness to the perfect concordance line (data accuracy) and the data’s tightness about its reduced major axis (the precision of the data) [48]. The CCC can essentially estimate the agreement between VACOD and FRCOD from a population causes of death level.

### Ethical approval

This study received the ethical approval to perform the VA interviews from the Institutional Review Board (IRB) at Makkah Health Affairs, Saudi Arabia (IRB Number: H-02-K-076-0321-478). The study was aligned with the Declaration of Helsinki. Formal verbal consent was obtained from all study participants before conducting the interviews, with the right to refuse or withdraw from interviews. Additionally, all data were collected and analyzed in accordance with the (IRB) ethical guidelines and regulations. While contact details of families of the deceased were initially provided for conducting the VA interview, any identifying information about the deceased or their families was removed from the data to ensure confidentiality and anonymity once the VA interview was completed. A newly encrypted data with anonymise IDs was produced to replace the original data prior to the analysis and storage.

The informed consent was documented using the consent form approved by the IRB. While this VA process took place at a distance via telephone calls, essentially to comply with the strict COVID-19 safety regulations at the time and to further reduce associated emotional distress, the VA interviewer demonstrated the contents of the consent form at the beginning of the VA interview, with a clear statement of the right of refusal or acceptance to entre or continue this VA interview. Upon declaration by the respondent, the VA interviewer registered the date, time, and state of the consent as approved (if the respondent agreed and continued until the end of the interview) or rejected (if the respondent disagreed to participate or declined to continue after initial agreement). All rejected interviews were discarded from the dataset and analysis. Since this is verbal consent, the VA interview assistant (FA) witnessed this consent process.

## Results

A total of 302 deceased relatives or caregivers were contacted and consented to conduct the VA interview, with three out of the respondents being female. Of these interviews, which were completed successfully over 4 months, InterVA-5 assigned either single, two, or three likely causes of death; a single cause of death to 299 individuals (99%), two likely causes to 26 individuals (8.60%), and three likely causes to 2 cases (0.66%). Three cases (0.99%) were explicitly designated ‘indeterminate’ by InterVA-5, generally reflecting a lack of particular VA data or contradictory evidence. Out of 302 VA interviews where FRCoD was reported, 142 families (47.01%) gave a single cause of death, and 124 families (41.05%) reported two causes. There were 36 families (11.92%) where the given likely causes of death were considered ‘indeterminate’ since they did not match with the International Classification of Diseases (ICD-10) codes.

**Fig 1:**
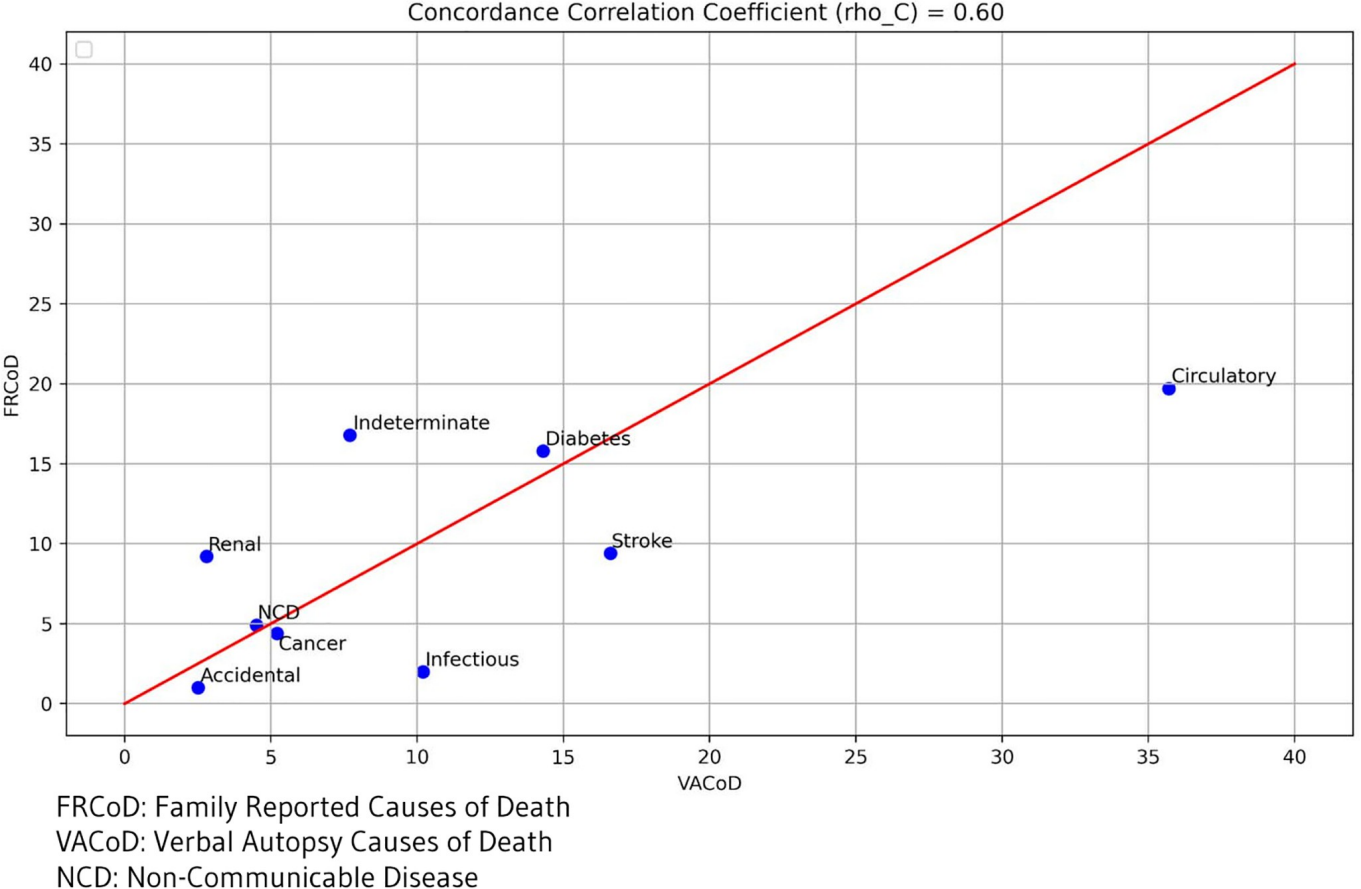
Concordance correlation coefficient (log-logscale) between CSMF determined by VA and respondent reports, in relation to the line of equivalence, for 302 deaths in Makkah city, Saudi Arabia.

Based on findings from the CCC, there was an overall agreement between (VACoD and FRCoD) in terms of cause-specific mortality. The Concordance of FRCoD concerning VACoD and fractions is shown in (Fig 1), with a CCC of 0.60 (95% CI: 0.20–1.00; p = 003).

To evaluate the community perceptions of causes of death, CSMFs obtained from the VA using the InterVA-5 model were utilized to assess FRCoD, along with the CSMF absolute differences and their 95% confidence intervals. Table 1 shows that infectious diseases, stroke, and circulatory-related deaths were found to be underestimated as probable causes of death reported by families in relation to the VA reference standard. In contrast, CSMF of indeterminate and renal-related deaths groups were overestimated as probable causes of death. In addition, accidental, NCD, and diabetes related deaths groups were slightly overestimated with a minimal absolute CSMF difference. Despite no ICD codes being assigned by InterVA-5 for COVID-19 (had no defined ICD code at the time) and mental illness-related deaths categories, the CSMF attributed to COVID-19 by families was as high as 13.18% of all deaths.

**Table 1:** Cause specific mortality fractions per disease categories derived from VACoD and FRCoD for 302 deaths, Makkah region, Saudi Arabia.

Categories	VACoD	FRCoD	Absolute Difference 95%CI
**Infectious**	10.2	2.0	-8.2 (-12.0,-4.4)*
**Cancer**	5.2	4.4	-0.8 (-4.2,2.6)
**Diabetes**	14.3	15.8	1.5 (-4.2,7.2)
**Circulatory**	35.7	19.7	-16.0 (-23.0,-9.0)*
**NCD**	4.5	4.9	0.4 (-3.0,3.8)
**Renal**	2.8	9.2	6.4 (2.6,10.2)*
**Accidental**	2.5	1.0	-1.5 (-3.6,0.6)
**Indeterminate**	7.7	16.8	9.1 (3.9,14.3)*
**Stroke**	16.6	9.4	-7.2 (-12.5,-1.9)*
**Covid-19**	0.0	13.1	13.2 (9.4,17.0)*
**Mental**	0.0	3.1	3.1 (1.1,5.1)*

**Abbreviation(s)**: FRCoD: Family reported causes of death.

VACoD: Verbal autopsy causes of death.

NCD: Non-Communicable disease.

*Significant Absolute Difference.

The results from a multivariable logistic regression model for exploring factors of determining the community perception are presented in Table 2. Based on the study findings, the odds ratio of the agreement decreases if the deceased was male compared to female (aOR: 0.52; 95% CI: 0.26–1.03) and it significantly increases if married compared to single (aOR: 2.13; 95% CI: 1.02–4.42). Improved perception of causes of death was associated with deaths of people with advanced education compared to illiterate (aOR:1.21; 95% CI: 0.45–3.20), and the death occurring at hospital compared to deaths at home (aOR: 1.25; 95% CI: 0.56–2.80), despite being not statistically significant. In addition, the odds of agreement tend to decrease if the interview was conducted with a respondent of a second-degree relationship to the deceased compared to a first-degree relationship (aOR: 0.91; 95% CI: 0.37–2.20). Our results found that the odd ratio of the agreement for age groups (60–69, 70–79, and > = 80 years) increases if the deceased aged 60–69 and 70–79 years compared to deaths aged 34–59 years, and the likelihood tends to decrease if the deceased was > = 80 years compared to the reference (aOR: 1.34; 95% CI: 0.60–2.98, aOR:1.04; 95% CI: 0.45–2.41 and, aOR:0.48; 95% CI: 0.16–1.38, respectively).

**Table 2:** Background and characteristics of 302 deaths in Makkah region, Saudi Arabia, and showing multivariable OR for agreement between FRCoD and VACoD.

Background characteristics	Number of deaths *n* (%)	Crude OR (95% CI)	Adjusted OR (95% CI)
**Age group**			
34–59	68 (22.52)	Ref	Ref. ^a^
60–69	88 (29.14)	1.37 (0.61–3.04)	1.34 (0.60–2.98)
70–79	84 (27.81)	1.09 (0.48–2.51)	1.04 (0.45–2.41)
> = 80	62 (20.53)	0.50 (0.17–2.42)	0.48 (0.16–1.38)
**Sex**			
Female	126 (41.72)	Ref	Ref. ^b^
Male	176 (58.28)	0.72 (0.40–1.31)	0.52 (0.26–1.03)
**Respondent**			
First degree relationship	260 (86.09)	Ref	Ref. ^c^
Second degree relationship	42 (13.91)	0.90 (0.37–2.16)	0.91 (0.37–2.20)
**Education**			
Illiterate	121 (40.07)	Ref	Ref. ^d^
Basic education	137 (45.36)	1.09 (0. 51–2.32)	1.09 (0.52–2.30)
Advance education	44 (14.57)	1.24 (0. 46–3.32)	1.21 (0.45–3.20)
**Year of death**			
2018	28 (9.27)	Ref	Ref. ^c^
2019	69 (22.85)	0.48 (0.14–1.54)	0.49 (0.15–1.62)
2020	161 (53.31)	0.90 (0.34–2.42)	0.90 (0.33–2.47)
2021	44 (14.57)	0.81 (0.24–2.66)	0.89 (0.26–2.99)
**Marital status**			
Single	116 (38.41)	Ref	Ref. ^c^
Married	186 (61.59)	1.78 (0.93–3.41)	2.13 (1.02–4.42)*
**Place of death**			
Home	60 (19.87)	Ref	Ref. ^c^
Hospital	242 (80.13)	1.29 (0.59–2.82)	1.25 (0.56–2.80)

**Abbreviation(s)**: FRCoD: Family reported causes of death

VACoD: Verbal autopsy causes of death.

^a^, adjusted for sex.

^b^, adjusted for education, marital status and age groups.

^c^, adjusted for age groups and sex.

^d^, adjusted for age groups, sex and marital status.

## Discussion

This study findings show a generally broad spectrum of community perceived mortalities with some crucial misconception based on the type of death and the background and characteristics of the deceased. During the data collection period, the world has experienced a devastating COVID-19 pandemic, which has ultimately re-profiled deaths and influenced the public and medical opinions of causes of death in Saudi Arabia and elsewhere. While the concept of community perception assessment is not new, understanding how local communities perceive cause of deaths, and consequently recognizing their associated risk factors is now timely and more crucial in view of the health and health system transitions due to COVID-19. Proposing such a standardized assessment would also be of interest to the Saudi health system to continuously monitor the health literacy of the people, particularly within such dynamic Saudi communities. Based on published reports, low health literacy–which is how people are able to access, understand, and utilize information and facilities to guide health-related decisions for themselves and other members in their communities–contributes to a wide range of health problems and is associated with poor health outcomes and ineffective chronic diseases management and treatments, such as the case of diabetes [19,50,51].

The study utilized VA to explore the community perception, which is mostly amenable for estimating CSMFs at population level and can adequately direct intended funds and interventional public health programs [52–54]. This study findings showed relatively minimal community misconception based on the derived CSMFs across diabetes, NCD, and accidental causes of death categories, implying that communities were more aware of and able to plausibly recognize these diseases as ultimate causes of death. Diabetes as a chronic illness demands frequent presentation at the healthcare services for routine follow up and treatments. High level of exposure to valid health information via professional medical practitioners can positively contribute to the community health literacy level, which may explain the higher level of perception for this cause of death category and likely other diseases from the NCD category. It can be said that communities are influenced by what they remember of signs and circumstances of death events, whereas other factors such as exposure to local beliefs or professional health practices can influence the subject’s memory [55]. For a disease such as cancer, for instance, mental representations of death can be exceptionally influential and highlight how communities may perceive some inevitable illnesses [56]. This study shows trivial CSMF differences due to cancer, where communities are arguably attributing cancer illness to death in a plausible manner [56,57]. Features of illness representations involve views about a disease’s etiology, time course, and curability [56].

For example, if individuals link cancer with death, they may avoid seeking health care services. In fact, there is considerable evidence that a fatalistic view of cancer hinders adherence to screening guidelines [58] and impedes engagement in cancer protective behaviours such as physical activity, healthy diet, and avoiding smoking [59,60]. Hence, addressing fatalistic beliefs through communication about certain diseases like cancer can play an important role in cancer prevention measures. Stroke as a cause of death, was however underestimated by communities, a pattern found to be consistent with previous Saudi and non-Saudi studies [61–63]. Difficulties in recalling or recognizing disease symptoms, rather than a mere lack of knowledge, can possibly explain the observed pattern by communities in relation to stroke, but may also reflect a need for a more effective stroke promotional campaign.

Public awareness of diseases that are associated with diabetes mellitus, like renal (kidney) and circulatory diseases, is important for public health interventional plans. Our results show that respondents tend to overestimate renal diseases as causes of death, and this pattern is consistent with prior cross-sectional research from Saudi Arabia [64,65], which revealed a dearth of knowledge about renal diseases among the Saudi population. While the prevalence of renal diseases is relatively high in Saudi Arabia due to climatic, behavioral, and genetic factors [66], the Saudi health system has a comprehensive treatment and follow up management, both in terms of dialysis services and kidney transplantation. This interaction between high disease prevalence and adequate healthcare and the fact that most of the study population are presumably diabetic (which is medically associated with renal diseases), seems to influence the community’s perception of causes of death related to renal diseases. Saudi communities have underestimated circulatory diseases’ cause of death, and this was also observed in other published reports revealing a low perception of cardiovascular causes of death [67–69]. This underestimation could be attributed to the disease complexity and general lack of information and awareness. Although this may seem plausible from a medical point of view, failing to recognize symptoms of common illnesses can have some public health implications since most deaths have taken place at hospital settings where relatives or caregivers are presumably informed about the illness of their deceased. This was also observed in other cross-sectional studies linking low health literacy skills with unrealistic risk underestimation [70,71]. Another plausible explanation is that circulatory diseases are frequently asymptomatic, with risk factors slowly progressing over time, leading to delayed recognition and perception. In line with a previous study [72], which demonstrated a low awareness of major infectious diseases, the present study revealed a low level of awareness in relation to infectious diseases, which is what would be expected in such setting due to stigmatization, and discrimination. This pattern supports the findings of previous studies in Saudi that reported a statistically significant positive correlation between some infectious diseases and stigma [73,74]. Stigma and discrimination in communities present a risk when reporting causes of death based on region, sex, and ethnicity, especially deaths related to stigmatized diseases [75]. As such, stigma can hinder accurate public awareness of some diseases and act as a barrier to people adopting health-promoting behaviors, seeking health care, and adhering to treatment.

The study findings indicate that community perception tends to be low if the deceased was > = 80 years compared to 34–59 years. Despite the association was lacking the statistical significance, this may be due to older people being more likely to develop chronic conditions, leading to disease recognition complexity at this age. Consequently, relatives attribute death to “fate “and perceive it as a natural part of the aging process rather than attributing it to a specific cause. We also found that community perception improves significantly if the deceased were married compared to single. Possibly, this is because deceased married individuals have a larger support network, including their spouse, family, and friends. Despite the ethical implications, symptoms and medical history of the deceased may have been shared with relatives, enabling a more accurate determination of the cause of death. Nevertheless, it should be noted that the results may vary according to the particular population being studied and the research methods used.

Although VA is commonly conducted in a registered population (a population being routinely monitored over time and space such as the concept followed in health socio-demographic and surveillance system), our study utilized medical records from the Saudi regional diabetes register as a denominator and later applied the VA process. Thus, the study findings may be overly pessimistic and have caused overlapping across the death categories. The method’s accuracy of cause of death determination is highly dependent on the type of death, the quality of the interview, and the procedures used to assign causes of death [34,76]. A variety of factors related to the interviewer, the respondent’s background, or both can impact the interview quality and thereafter the VA results. The method has been shown to be reasonably practical for identifying causes of death in infancy or due to specific conditions such as injuries or maternal causes. On the other hand, medical causes of adult deaths share symptom complexes, making it difficult to differentiate between various causes of death from such descriptions when based solely on symptoms provided by relatives or occasionally by non-relative such as employers or work colleagues, mainly in deaths among expats. Subjects with T2DM, for example, have a two- to four-fold increased risk of myocardial infarction and sudden death compared to individuals without diabetes, whereby roughly half of all diabetes deaths co-exist with some types of cardiovascular diseases [77]. This demonstrates the disease’s complexities and how the community may fail to recognize the true causes of death without effective health promotion and educational programs.

Despite the use of an internationally recognized standard VA method, interpreting and generalizing such findings in view of the small sample size can be challenging. The VA telephone interviews were used instead of the traditional face-to-face method to comply with the imposed COVID-19 pandemic’s physical distancing measures at the time of the data collection. Nevertheless, numerous studies have found that telephone interviews are as effective as face-to-face interviews [78,79]. According to one study, the results of VA interviews via phone calls were consistent with previous literature, which found that the telephone interview method to be feasible, accepted by caregivers and healthcare workers, and has a reliable level of data quality [80]. One potential issue with VA interviews is the risk of selection bias. This can result from inaccuracies in the recorded contact details. Another contributing factor was respondent reluctance (i.e. refusing to participate or withdraw from the interview). Furthermore, language can be a factor, as we only interviewed individuals proficient in Arabic or English to ensure the interview’s quality. Given the ethical nature of VA, it can arguably be said that conducting distant VA interviews on a fairly small sample in an area where VA has never been practiced before can be viewed as a strength in this study from an ethical perspective, which has the potential of reducing unwanted emotional or cultural consequences.

## Conclusion

Saudi community perceptions of causes of death with reported T2DM was relatively plausible but varied substantially based on the type of death, and other vital events like marital status. Applying VA within medical services in the Saudi context will likely generate a meaningful public health implication of how people may perceive risk factors associated with the causes of death, which can inform targeted public health intervention programs. This highlights the importance of integrating rigorous and consistent VA methods within the routine medical services rather than relying on individual opinions mainly for complex causes of death outcomes. Nevertheless, translating cause-specific mortality data into effective policymaking remains challenging without standardized assessment of non-medical causes such as social and health system aspects contributing to death.

## Supporting information

S1 Table Categorizing of causes of death reported by VA and families. (PDF)
