# Supplementary material for: Community perception of causes of death using verbal autopsy for diabetes mellitus in Saudi Arabia
Source: PLOS Glob Public Health. 2023 Dec 5;3(12):e0001690. doi: 10.1371/journal.pgph.0001690 (PMC10697554; doi:10.1371/journal.pgph.0001690)
Supplement: S1 Table — (PDF) [file pgph.0001690.s001.pdf]

**S1 Table. Categorizing of causes of death reported by VA and families.**

| Category | Cause of death |
|---|---|
| Infectious diseases | Sepsis (non-obstetric)<br>Acute resp infect incl pneumonia<br>HIV/AIDS related death<br>Pulmonary tuberculosis<br>Other and unspecified infect diseases |
| Cancer | Digestive neoplasms<br>Respiratory neoplasms<br>Breast neoplasms<br>Other and unspecified neoplasms<br>Pancreatic cancer, Lung cancer<br>Tumors, Head cancer |
| Diabetes mellitus | Diabetes mellitus<br>Gangrene, Diabetic foot amputation, foot ulcer |
| Circulatory diseases | Acute cardiac disease<br>Other and unspecified cardiac disease<br>Heart failure, heart attack cardiomyopathy or myocardial insufficiency<br>Cardiac arrest, Heart attack, Hypertension, Hypotension, Heart disease |
| Non-Communicable disease. | Chronic obstructive pulmonary disease<br>Abdominal related<br>Acute abdomen<br>Other and unspecified NCD |
| Renal diseases | Kidney disease, kidney failure, dialysis<br>Renal failure |
| Accidental | Accidental fall<br>Road traffic accident<br>Accidental injuries<br>Accidental expos to smoke fire & flame |
| Indeterminate | Any cause of death that has no ICD code |
| Stroke | Stroke |
| Covid-19 | Covid-19 |
| Mental illness | Dementia, Alzheimer |
